# The evolution of ciliary signaling mechanisms

**DOI:** 10.1186/2046-2530-1-S1-P11

**Published:** 2012-11-16

**Authors:** M Abedin, JFR Reiter

**Affiliations:** 1 University of California, San Francisco, USA

## 

Cilia, evolutionarily ancient structures that project from the cell surface into the extracellular environment, are critical sensors of external cues. In animals, they detect light, odors, soluble chemicals and mechanical forces, and defects in cilia cause a range of diseases. Vertebrate cilia respond to intercellular signaling molecules such as Hedgehog, a regulator of embryonic development. Despite its important roles in embryogenesis and disease, little is known about the evolutionary origins of ciliary signaling. Understanding how cilia evolved mechanisms to receive and transduce signals will help identify core ciliary functions fundamental to animal biology. To shed light on the evolution of ciliary signaling, I will characterize the signaling function of cilia in a close relative of animals, choanoflagellates. Choanoflagellates provide a simple biological system in which to elucidate mechanisms of ciliary signaling and identify signaling molecules that may be conserved in mammals. I will address two questions: (1) What signaling proteins are present in choanoflagellate cilia? And (2) what are the functions of choanoflagellate homologs of Hedgehog pathway components? I will isolate cilia from choanoflagellates to identify putative ciliary signaling proteins using mass spectrometry and carry out follow-up studies on candidate proteins prioritized based upon their homology to animal proteins. To understand the ancestry of a known ciliary signaling pathway, I will characterize choanoflagellate homologs of the Hedgehog pathway members Patched (receptor) and Hedgehog (ligand) using a heterologous expression system. This research will provide novel insights into the evolution of ciliary signaling and mechanisms of disease-associated signal transduction.

